# Thematic Analysis of Home Alone: An Intervention for Older Adults Living Alone With Memory Impairment

**DOI:** 10.1093/geroni/igaf122.2841

**Published:** 2025-12-31

**Authors:** Grace Savard, Robyn Birkeland, Amy Hobday, Stephanie Ingvalson, Laura Gitlin, Renee Pepin, Jill Cigliana, Joseph Gaugler

**Affiliations:** University of Minnesota Twin Cities, Minneapolis, Minnesota, United States; University of Minnesota, Minneapolis, Minnesota, United States; University of Minnesota, Minneapolis, Minnesota, United States; University of Minnesota, Minneapolis, Minnesota, United States; Drexel University, Philadelphia, Pennsylvania, United States; Geisel School of Medicine at Dartmouth, Lebanon, New Hampshire, United States; Memory Care Home Solutions, St. Louis, Missouri, United States; University of Minnesota, Minneapolis, Minnesota, United States

## Abstract

Older adults who live alone with mild cognitive impairment (MCI) or subjective memory impairment (SMI) are at greater risk for loneliness, safety concerns, and physical and mental health concerns. These risks can lead to an increased need for support, supervision, and potentially residential care. Interventions are needed to reduce the risk of isolation and adverse health outcomes for older adults with MCI or SMI. Home Alone, a novel psychoeducational program, includes home safety education and utilizes Behavioral Activation to improve socialization and engagement among older adults with MCI or SMI who live alone. The seven-session intervention is delivered by a trained coach visiting the participant’s home or via telehealth. Session foci include home safety, resource provision, motivational strategies, memory aids, and an in-depth exploration of personal values, followed by identification and scheduling of values-aligned activities. Sixty-five participants enrolled across Phase I (feasibility study, n = 15) and Phase II (pilot test of revised Home Alone intervention, n = 50) of the Home Alone study. Qualitative data were collected from participants via semi-structured interviews and open-response items on two sequential follow-up surveys. Thematic analysis revealed how the Home Alone program raised awareness and influenced engagement in socialization and meaningful activity, increased utilization of resources and daily living strategies, and prompted home safety modifications. Analyses provided nuanced insights into Home Alone’s impacts on participant well-being while shedding light on ‘program fit’ and participant concerns for the future. These findings will inform subsequent Home Alone analyses and eventual dissemination of the intervention to community settings.

